# Warburg’s vision

**DOI:** 10.7554/eLife.29217

**Published:** 2017-07-06

**Authors:** James B Hurley

**Affiliations:** 1 Department of Biochemistry, University of Washington, Seattle, United Statesjbhhh@uw.edu; 2 Department of Ophthalmology, University of Washington, Seattle, United States

**Keywords:** Warburg effect, outer segments, retinal metabolism, allostery, Mouse

## Abstract

Genetic tools help to dissect the relationship between aerobic glycolysis and anabolic metabolism in the retinas of mice.

**Related research article** Chinchore Y, Begaj T, Wu D, Drokhlyansky E, Cepko CL. 2017. Glycolytic reliance promotes anabolism in photoreceptors. *eLife* **6**:e25946. doi: 10.7554/eLife.25946

Rod-shaped cells at the back of our eyes allow us to see in dim light. Each day, around dawn, these rod cells shed their tips (LaVail, 1976), and the lost material is replaced with newly built proteins and other macromolecules made further down in the same cell (Anderson et al., 1980; Young, 1967).

Cancer cells growing in a tumor also have a high demand for newly built macromolecules. In the early 1920s, the German physiologist Otto Warburg reported on a specialized type of metabolism that converts most of the glucose taken up by a cell into lactate, rather than carbon dioxide and water as usually happens, even when oxygen is abundant (Warburg et al., 1924). Two of the tissues in which Warburg discovered this type of metabolism, which is often referred to as "aerobic glycolysis", were the very same tissues introduced above, retinas and tumors.

The building of complex macromolecules from simpler building blocks is referred to as anabolism. Recently studies into the metabolism of cancer cells have begun to reveal biochemical details that may link aerobic glycolysis and anabolic activity (Vander Heiden and DeBerardinis, 2017). One of the remarkable features of cancer cells discovered in these studies is that they often produce specific versions (or isoforms) of the enzymes that carry out glycolysis, namely pyruvate kinase (PKM2) and lactate dehydrogenase (LDHA). Not surprisingly, these same isoforms are present in rod cells (Casson et al., 2016; Lindsay et al., 2014; Rajala et al., 2016; Rueda et al., 2016). Now, in eLife, Constance Cepko and colleagues at Harvard Medical School – including Yashodhan Chinchore as first author – report how these two glycolytic enzymes contribute to anabolic metabolism in rod cells from mice (Chinchore et al., 2017).

First, Chinchore et al. inactivated LDHA and PKM2 in mouse rod cells, either with inhibitors or by reducing expression of the genes that encode the enzymes. The resulting rod cells were shorter than normal, as if they did not have enough anabolic activity to counteract the shedding of their tips (Figure 1). In support of this idea, when the mice were kept in constant darkness (which suppresses shedding and renewal of the outer segments), inactivating LDHA or PKM2 had less of an effect. Chinchore et al. then engineered mice in which some cells in the retina made less LDHA or PKM2 while the others were normal. In these 'mosaic' retinas, the only rods that were shorter were the ones with less LDHA or PKM2. This suggests that the enzymes promote anabolism only in the cell in which they are made.

**Figure 1.**
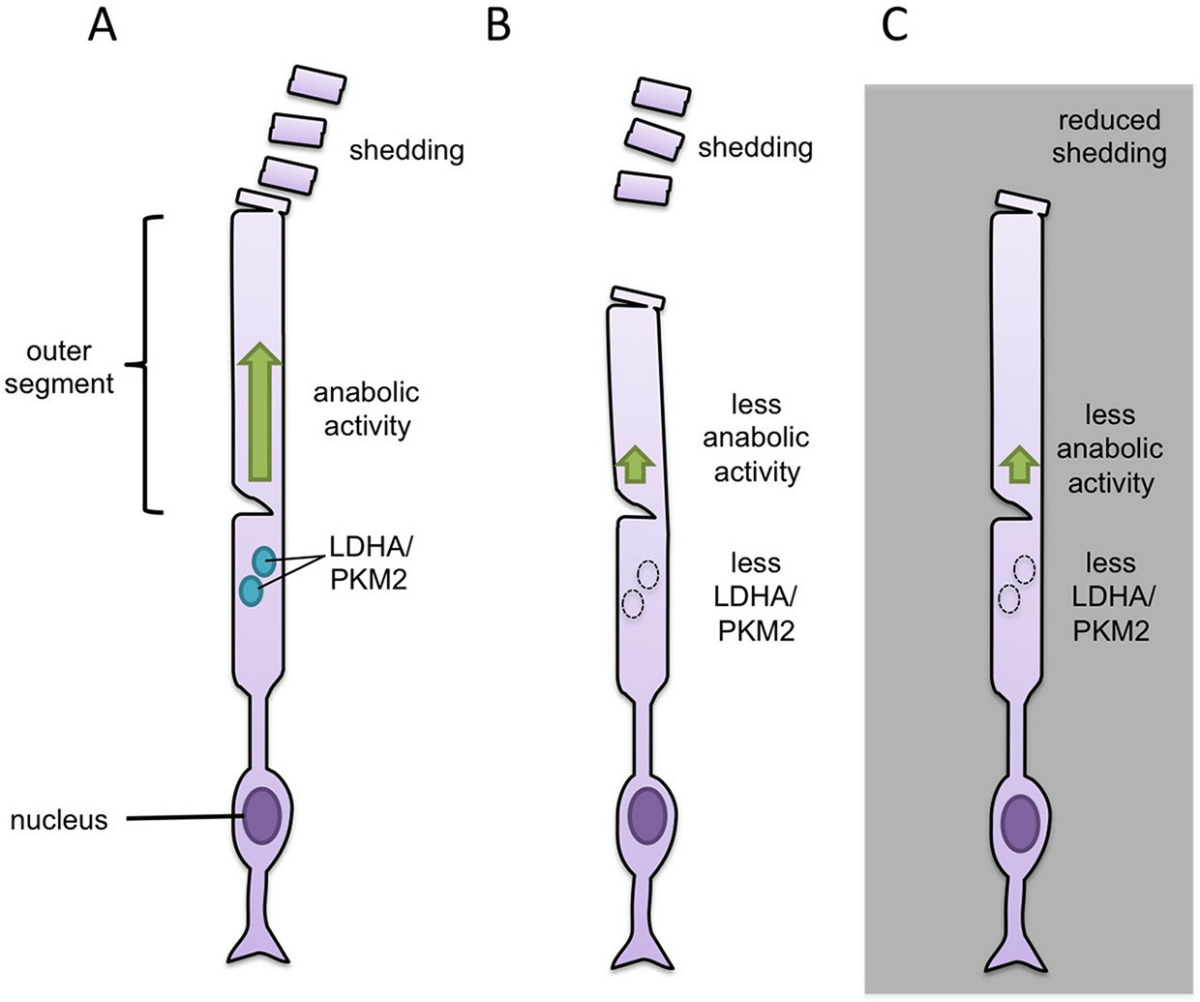
Manipulation of glycolytic enzymes sheds light on the link between aerobic glycolysis and anabolic metabolism. (**A**) The tip of the outer segment of a rod cell in the retina regularly sheds and needs to be replaced. Anabolic activity (green) in the same cell builds the macromolecules needed to replace the lost material. Two glycolytic enzymes, called PKM2 and LDHA (light blue circles), are thought to drive the anabolic metabolism of rod cells. (**B**) Reducing the expression of either of the genes for these enzymes results in rod cells with shorter outer segments, most likely because there is not enough anabolic activity to counteract the shedding. (**C**) Keeping mice in constant darkness suppresses the shedding and means that rod cells that lack LDHA or PKM2 still have outer segments of a normal length.

Chinchore et al. were concerned that the complete loss of PKM2 or LDHA might have effects that were so devastating that even the essential 'housekeeping' roles of glycolysis in the cell could be compromised. To address that concern, they also used a more 'surgical' approach that specifically slowed glycolysis without eliminating the entire pathway. Fructose-2,6-bisphosphate is an activator of glycolysis. To decrease this chemical in rod cells, Chinchore et al. overexpressed a protein specifically in rods that removes an essential phosphate group from this activator. Slowing glycolysis by this strategy made the rod cells shorter than normal, indicating that flux through the glycolysis pathway is key.

So, what makes PKM2 and LDHA different from other isoforms so that cells requiring rapid growth use them and not the other isoforms? Previous studies showed that these enzymes can be regulated by tyrosine phosphorylation to promote aerobic glycolysis (Hitosugi et al., 2009; Jin et al., 2017). Moreover, exposure to light – which increases the need for anabolic activity – enhances phosphorylation of PKM2 in the retinas of mice (Rajala et al., 2016). Chinchore et al. confirmed this result and then looked for signaling pathways that, when blocked, reduced how much PKM2 was phosphorylated in rod cells. They found that the pathway that responds to fibroblast growth factor (FGF) can control the phosphorylation of PKM2 and LDHA in mouse retinas. The tissue that normally is immediately adjacent to the rod cells, the retinal pigment epithelium, can influence the amount of FGF that a retina is exposed to in an eye. Chinchore et al. found that culturing mouse retinas with this tissue, or with some FGF, boosts how much lactate is produced.

By manipulating the expression of genes involved in aerobic glycolysis in mouse retinas, Chinchore et al. have further revealed how aerobic glycolysis relates to anabolic metabolism. They also show that disrupting any of three different steps in glycolysis can diminish anabolic capacity and cause the rod cells to become shorter. Each of the disruptions tested would have a different biochemical effect on the glycolytic pathway, but what they have in common is that they all cause less lactate to be produced. It is not yet clear why this would compromise anabolic activity, but the genetic tools developed by Chinchore et al. to manipulate glycolysis in rod cells provide new opportunities to answer this question.
